# Interoceptive accuracy is associated with benefits in decision making in children

**DOI:** 10.3389/fpsyg.2022.1070037

**Published:** 2023-01-19

**Authors:** Olga Pollatos, Karla Mönkemöller, Karoline Groppe, Birgit Elsner

**Affiliations:** ^1^Clinical and Health Psychology, Institute of Education and Psychology, Ulm University, Ulm, Germany; ^2^Evangelisches Krankenhaus Königin Elisabeth Herzberge, Berlin, Germany; ^3^Department of Psychology, Faculty of Human Sciences, University of Potsdam, Potsdam, Germany

**Keywords:** cardiac perception, interoception, emotion, decision making, Iowa gambling task, somatic-marker hypothesis, childhood development

## Abstract

**Introduction:**

Decision making results not only from logical analyses, but seems to be further guided by the ability to perceive somatic information (interoceptive accuracy). Relations between interoceptive accuracy and decision making have been exclusively studied in adults and with regard to complex, uncertain situations (as measured by the Iowa Gambling Task, IGT).

**Methods:**

In the present study, 1454 children (6-11 years) were examined at two time points (approximately 1 year apart) using an IGT as well as a delay-of-gratification task for sweets-items and toy-items. Interoceptive accuracy was measured using a child-adapted version of the Heartbeat Perception Task.

**Results:**

The present results revealed that children with higher, as compared to lower, interoceptive accuracy showed more advantageous choices in the IGT and delayed more sweets-items, but not toy-items, in a delay-of-gratification task at time point 2 but not at time point 1. However, no longitudinal relation between interoceptive accuracy and decision making 1 year later could be shown.

**Discussion:**

Results indicate that interoceptive accuracy relates to decision-making abilities in situations of varying complexity already in middle childhood, and that this link might consolidate across the examined 1-year period. Furthermore, the association of interoceptive accuracy and the delay of sweets-items might have implications for the regulation of body weight at a later age.

## 1. Introduction

To decide in favor of positive outcomes in the future rather than of short-term benefits accompanied by long-term disadvantage is a common and crucial challenge of daily life. Impaired decision making has been related to a diverse range of problems, such as impulsivity (e.g., Franken et al., 2008), drug use (e.g., Grant et al., 2000), or extreme weight conditions and eating disorders (e.g., Brogan et al., 2010). Considering multiple alternatives and reflecting about future consequences has long been seen as a pure rational function. However, there is evidence that decision making is not only a result of logical analyses, but is further guided by somatic information (e.g., Werner et al., 2009).

Research on this topic has mainly been based on the somatic-marker hypothesis (SMH) proposed by Damasio (1994, 1996), which builds on earlier work linking bodily activity to the experience of emotion (e.g., James, 1884) and to decision making (e.g., Nauta, 1971). The SMH suggests that somatic information assists decision making, especially in complex or uncertain situations. More specifically, in such situations each response option should be associated with specific learned somatic responses (e.g., heart rate, skin conductance, tonicity) that either encourage or discourage the particular option. These so-called somatic markers are thought to be represented and regulated in secondary representation areas, in particular the ventromedial prefrontal cortex (VM-PFC; Damasio, 1996; Bechara et al., 2000) or the anterior cingulate (ACC; Bechara and Naqvi, 2004).

Evidence for the SMH is mainly based on adults’ performance in the Iowa Gambling Task (IGT), which has been designed in order to mimic real-life decision-making, thereby incorporating factors like uncertainty, reward, and punishment. For a successful performance on this task, participants have to learn during the course of trials to forgo short-term benefit for long-term profit while choosing between alternatives varying in pay-off and punishment. More precisely, they need to draw cards selecting between card decks which result in larger gains but also in high unpredicted losses, and decks which result in smaller gains, similarly small losses, and an overall net profit (Bechara et al., 1994).

According to the SMH, individuals who ascribe more precision to signals from the viscera should show superior performance in this task. Indeed, a successfully learning to choose the advantageous decks (with smaller gains, but also smaller loss) in the IGT has been linked to somatic-marker signals, indexed by anticipatory skin conductance responses. For example, adult patients with lesions in the VM-PFC do not seem to develop somatic markers in the form of skin-conductance responses and continue to choose disadvantageous options during the IGT, indicating that they are driven more by the immediate reward than by future consequences (“myopia for the future”; Bechara et al., 1996, 1997; see Dunn et al., 2006 for a review).

To date, most research on the SMH has rarely used other sources of bodily feedback except from skin-conductance responses (Dunn et al., 2006). However, cardiac cues should just as well function as a potential somatic marker guiding decision making. Individual differences in perception of and sensitivity to changes within the internal bodily state are one way of quantifying the access to bodily cues (Herbert and Pollatos, 2008). Research regarding interoception has mainly focused on the ability to detect cardiovascular signals (e.g., Critchley et al., 2004; Pollatos et al., 2005), mostly quantified in heartbeat-perception tasks. Results underscore that there are significant interindividual differences in adults’ cardiac perception, being interpreted as trait-like sensitivity toward one’s visceral signals, which is understood as a long-term result of “visceral” learning processes. Such processes depend on autonomic reactivity during different situations of daily life that evoke substantial changes in autonomic activity (Herbert and Pollatos, 2012; Herbert et al., 2013).

Garfinkel et al. (2015) proposed a differentiation between three separable dimensions of interoception, namely interoceptive accuracy (IAcc; i.e., performance on objective behavioral tests of heartbeat detection), interoceptive sensibility (i.e., self-evaluated assessment of subjective interoception) and interoceptive awareness (i.e., metacognitive awareness of interoceptive accuracy). More recently, this differentiation was extended by Pollatos and Herbert (2018) who suggested interoceptive emotional evaluation (i.e., interpretation of perceived bodily signals) as a fourth dimension of interoception.

To our knowledge, only a few studies have examined associations between interoceptive accuracy and decision making, and these studies report mixed results. Werner et al. (2009) found that adult individuals with particularly accurate cardiac-perception ability show superior decision-making performance in the IGT compared to individuals with low perception accuracy, but these results were not replicated in a more recent publication (Werner et al., 2013). Werner and colleagues attributed this to the small number of participants with high interoceptive accuracy. Furthermore, in a refined version of the IGT, interoceptive accuracy was associated with either good or poor decision-making performance, depending on whether anticipatory bodily signals favored advantageous or disadvantageous choices (Dunn et al., 2010). In a modified Go-No Go paradigm, not interoceptive accuracy but interoceptive awareness was related to voluntary inhibition decisions, such that subjects with lower awareness of bodily signals were more likely to act and respond faster when they had the choice (Rae et al., 2020). Moreover, Wölk et al. (2014) showed that higher interoceptive accuracy is related to improved decision making only in healthy participants, but to impaired performance in patients with panic disorder. Accordingly, impaired decision-making performance on an IGT was found to be predicted by diminished interoceptive accuracy in individuals with gambling disorder (Moccia et al., 2021) and alcohol use disorder (Avcu Meriç and Sönmez, 2022). Sugawara et al. (2020) identified a positive link between the shift toward rationality in a decision-making task and the improvement of interoceptive accuracy after an interoceptive training. This relation was further demonstrated to have an affective dimension as well: Sokol-Hessner et al. (2015) identified interoceptive accuracy to predict aversion to loss in a gambling task.

So far, the role of somatic markers in decision making has been investigated exclusively in adult populations. Thus, it remains unclear whether this association is already present in children or whether it arises later in the course of development. There is evidence that already children differ in their ability to perceive ongoing signals deriving from the heart, and that this ability might function as a basis for their emotional experience in similar ways as it has been reported for adults (Koch and Pollatos, 2014a). Crone and van der Molen (2007) found that the autonomic bodily processing of decision outcome is similar across age groups (8–10, 12–14, and 16–18 years of age) in children. Furthermore, the role of interoceptive development was emphasized in the context of mental health and pain: Hechler (2021) postulates a bilateral relation between interoceptive processes on the one hand and the genesis of mental health problems and chronic pain on the other hand: in a complex framework including physiological, cognitive and emotional aspects, somatic responses potentially contribute to the co-occurrence of mental health problems *via* interoceptive fear conditioning. Opdensteinen et al. (2021) identified a positive relation between interoceptive accuracy and emotion regulation, another important aspect of executive functioning, in a sample of preschool children. However, it remains an open question whether the access to these bodily signals (i.e., interoception) influences decision making in children. Furthermore, it has been found that executive function, which as a higher-level construct includes decision-making abilities, improves most rapidly during the preschool period, but continues to develop during middle childhood and adolescence. Such developmental changes in executive function are related to substantial structural and functional changes in neural systems involving the PFC (Pennington and Ozonoff, 1996; Zelazo et al., 2008; Hughes, 2011). Thus, the first aim of the present study was to examine whether children with higher (as compared to lower) interoceptive accuracy show better decision-making (i.e., more advantageous choices) as early as in middle childhood, when both abilities have presumably not yet been finally developed.

Research on the role of somatic markers in decision making has exclusively focused on variants of the IGT, which measures intuitive decision-making patterns given incomplete information or ambiguous consequences, respectively. Because originally, the implicit nature of somatic markers has been emphasized, the IGT was designed to study decision making in complex situations that cannot completely be captured by reflective knowledge alone (Dunn et al., 2006). Another ability related to affective decision making and also regulated by the VM-PFC is delay of gratification (DoG), which is usually assessed by tasks requiring a choice between receiving a smaller reward immediately or a more valuable reward later on (Happaney et al., 2004; Hongwanishkul et al., 2005). Whereas both the IGT and DoG tasks require deciding in favor of an advantageous outcome in the future, they differ with respect to the time that children need to wait for rewards, and to the certainty with which rewards are obtained: choice contingencies remain purposely unclear in the IGT, but are clearly stated in DoG tasks, making the latter less ambiguous and complex (Hongwanishkul et al., 2005). In adults, there is evidence that affective somatic markers such as mood might influence impulsive behavior in tasks that require delaying a reward in order to optimize the outcome (Lerner et al., 2013; Weafer et al., 2013; for review see Herman et al., 2018), indicating the relevance for a DoG task in research on the SMH and interoception. Thus, our second aim in the present study was to explore whether interoceptive accuracy is also related to children’s performance in a DoG task, that is, in decision-making processes that are emotionally or motivationally relevant, but less complex than those elicited in the IGT.

From a developmental perspective, it would furthermore be interesting to address the direction of effect between interoceptive accuracy and decision making. To date, however, longitudinal studies are lacking. Because higher interoceptive accuracy relates to an enhanced central-nervous-system processing of bodily signals (Dunn et al., 2010; Werner et al., 2013), it can be assumed that a higher interoceptive accuracy in the long term leads to a behavioral advantage in situations generating somatic-marker signals. Thus, a better perception of and higher sensitivity to somatic-marker information should lead to an advantage in the development of decision-making abilities in situations that are emotionally or motivationally relevant.

To sum up, we examined a large sample of children between 6 and 11 years of age and expected children with higher interoceptive accuracy to opt for more advantageous options or learn to do so during the course of several trials in a child-adapted IGT (research question 1), and also to opt for more advantageous choices in a DoG task (research question 2), as compared to children with lower interoceptive accuracy. We examined these hypotheses at two measurement time points, about 1 year apart, in order to detect developmental differences in the relation of interoceptive accuracy and decision making. Moreover, we expected level of interoceptive accuracy to function as a longitudinal predictor of IGT and DoG performance 1 year later (research question 3).

## 2. Materials and methods

### 2.1. Participants and procedure

Data for this study was collected within a large longitudinal study on intrapersonal developmental risk-factors in childhood and adolescence (PIER study), which has started in 2012. Data on the PIER study has already been reported elsewhere using the same measures as the present study while focusing on different research questions (Groppe and Elsner, 2014, 2015, 2017; Koch and Pollatos, 2014a,b). The first two assessments of the PIER study were separated by a time interval of approximately 1 year (*M* = 273 days, *SD* = 55 days).

At the first measurement time point (t1), a total of 1,658 children (52.1% girls) aged 6 to 11 years were recruited from 33 elementary schools from the federal state of Brandenburg (German school classes 1–3). Schools were preselected for a representative variety of social backgrounds, both urban and rural. At the second time point (t2), 1,619 of these children now aged 7 to 11 years took part. The samples used in the present analyses consisted of 1,446 children at t1 (*M* age = 8.4 years, *SD* = 0.95; 51.9% girls) and 1,454 children at t2 (*M* age = 9.1 years, *SD* = 0.92; 51.7% girls) who provided data on the Heartbeat-Perception Task. Missing data was mainly due to technical problems leading to invalid data on this task.

At each measurement time point, children were tested individually with regard to various psychological variables by a trained and supervised doctoral student or research assistant. Testing took place during the morning hours in a quiet room either at school or at home on 2 days within 1 week. The order of tasks was counterbalanced across participants (blocks of ABCD/BADC). Subsequent analyses, however, revealed no effect of task sequence. Informed consent was obtained for each child from a primary caregiver, and the children received a cinema voucher for their participation at both time points. Approval for the study was obtained by the Research Ethics Board at the University of Potsdam and by the Ministry of Education, Youth and Sports of the Federal State of Brandenburg.

### 2.2. Materials

All measures were obtained at both t1 and t2. Because measures were identical to those used in our previous studies (see Groppe and Elsner, 2014, 2015, 2017; Koch and Pollatos, 2014a,b), we will only give a short synopsis at this point.

In order to measure complex decision making, we used a slightly modified version of the computer-based Hungry Donkey Task (Crone and van der Molen, 2004), which is a child-adapted version of the IGT (Bechara et al., 1994). Children were asked to assist a hungry donkey in collecting as many apples as possible across 60 trials. Furthermore, participants were told that they could win a marble if they collected at least 20 apples. Each trial consisted of pressing 1 of 4 keys, each opening a corresponding door (A, B, C, D) that appeared side by side on a computer screen. Upon pressing a key, an outcome display indicated the number of apples gained (in green) and/or lost (in red), as well as the overall sum of gained and lost apples across previous trials. Selecting doors A or B resulted in larger gains but also in high unpredicted losses, leading to an overall net loss of 10 apples per 10 trials. Selecting doors C or D resulted in smaller gains but similarly small losses and an overall net profit of 10 apples per 10 trials. Usually, participants start the task by choosing doors more or less randomly, followed by an increasing preference for the advantageous doors (C, D; e.g., Crone et al., 2005). As dependent variables, we used the number of advantageous doors selected. For the analyses of research questions 1 and 2, the number of advantageous doors selected was broken down into 6 blocks of 10 trials in order to depict potential learning effects over time (see Crone and van der Molen, 2004).

To measure delay of gratification (DoG), we asked children to choose between receiving a smaller reward immediately or a larger reward 1 week later (which they would actually get at the second test session; adapted from Wulfert et al., 2002). There were 4 trials, in which the child always saw the immediate (smaller) reward and was verbally informed about the delayed (larger) reward that always consisted of more items of the same type. Two trials contained sweets-items (immediate vs. delayed: 1 vs. 2 chocolate drops; 1 vs. 5 chewing candies) or toy-items (1 vs. 2 bouncing frogs, 1 vs. 3 tattoos), respectively. As dependent variables, we used the number of trials (0 to 2) in which the child chose to delay sweets or toys. As this variable reflects the actual number of delayed items, it is a ratio variable with adequate scaling for the subsequent analysis. The order of item-presentation (alternating between sweets and toys) was counterbalanced across participants (with two different sequences). Subsequent analyses, however, revealed no effect of item sequence. In a pretest (Groppe and Elsner, 2014) on 41 children who did not participate in the present study (*M* age = 8.41, *SD* = 0.49; 54% girls), the number of delayed trials showed positive associations in the medium range (*r* = 0.31–0.37, *p* ≤ 0.05) with impulsivity (German version of Eysenck’s I6 Impulsivity Scale; Stadler et al., 2004), delay-of-gratification in eating (subscale from the Delaying Gratification Inventory; Hoerger et al., 2011) and academic delay-of-gratification (Academic Delay of Gratification Scale for Children; Zhang et al., 2011). These results suggest good convergent validity of our DoG measure. Furthermore, in the pretest, the four trials used in the present study were highly associated with an 8-item version of the DoG task (*r* = 0.88, *p* < 0.001).

Interoceptive accuracy was measured by the Heartbeat-Perception Task, following the mental-tracking method proposed by Schandry (1981) and adapted for children (see Koch and Pollatos, 2014a). The original interval length was shortened, resulting in three fixed intervals of 15, 20, and 18 s, plus a short initial training interval of about 10 s. During each interval, children were seated and told to silently count their own heartbeats without feeling for their pulse and without trying to facilitate heartbeat detection by physical manipulations (e.g., holding their breath). Simultaneously, cardiac activity was recorded noninvasively using the mobile heart-frequency monitor RS800CX (Polar Electro Oy, Kempele, Finland), which has shown equally good validity and reliability as compared to alternative ECG measurement devices in populations of children and adults (e.g., Kingsley et al., 2005; Gamelin et al., 2008; Nunan et al., 2008). The electrode strip was attached to both hands and affixed to a table. Signals were sampled at 1,000 Hz and analyzed by the Polar ProTrainer 5 software (version 5.40.172), which is based on the HRV analysis software of the University of Kuopio, Finland (Niskanen et al., 2004). Heartbeat-perception scores were calculated taking the mean difference between recorded and counted heartbeats across the three intervals (1/3 ∑ [1 − (|recorded heartbeats − counted heartbeats| /recorded heartbeats)]). According to this formula, higher scores indicate a higher interoceptive accuracy with a maximum score of 1 (absolute accuracy) and a minimum score of 0 (child did not perceive any heartbeat). The internal consistency of the task was excellent at both measurement time points (Cronbach’s α t1: 0.91, t2: 0.90).

### 2.3. Statistical analyses

We divided the sample into children with higher versus lower interoceptive accuracy scores using a median-split (Mdnt1 = 0.59; Mdnt2 = 0.59). The two groups were compared using a two-tailed unpaired *t*-test. Additionally, intercorrelations between the distinct variables at both time points were explored. Analyses for the first two research questions were conducted separately at both measurement time points in order to detect possible developmental changes.

In order to answer our first research question (whether children with higher as compared to lower interoceptive accuracy opt for more advantageous options in the IGT, or learn to do so across the course of trials) we calculated separate ANOVAs at t1 and t2 on the mean number of advantageous doors (C, D) selected, with the within-subjects factor Trial Block (1–6) and the between-subjects factor Interoceptive Accuracy (higher vs. lower). Subsequently, this analysis was repeated across distinct age groups in order to gain closer insights in the development of the described abilities. Because Mauchly’s test indicated some violations of the assumption of sphericity, the respective degrees of freedom were corrected using Greenhouse–Geisser estimates. For each analysis, children with complete data on all variables of interest were included. Thus, the number of children slightly varies for the three hypotheses depending on the examined variables.

For our second research question (whether children with higher as compared to lower interoceptive accuracy opt for more advantageous options in the DoG task) we conducted separate MANOVAs at t1 and t2 on the number of toy-items delayed and the number of sweets-items delayed, with the between-subjects factor Interoceptive Accuracy (higher vs. lower).

For our third research question (whether interoceptive accuracy longitudinally predicts later performance in the IGT and DoG task) we calculated first a regression of IGT performance t2 (overall score across trials 1–60) on interoceptive accuracy t1, controlling for age and IGT performance at t1, and second a regression of DoG performance t2 (separately for toy- and sweets-items) on interoceptive accuracy t1, controlling for age and DoG performance at t1. We treated interoceptive accuracy t1 again as a dichotomous variable to allow for a comparability of results.

## 3. Results

Bivariate correlations, as well as means and standard deviations, for all of the assessed variables are summarized in Table 1. In general, correlations of assessed variables and age were low or non-significant. Furthermore, the two groups of children differed significantly with regard to their interoceptive accuracy at t1 [*M*_lower IAcc_ = 0.34, *SD* = 0.19; *M*_higher IAcc_ = 0.76, *SD* = 0.10, *t*(1121.49) = −52.89, *p* < 0.001, *r* = 0.15] and t2 (*M*_lower IAcc_ = 0.36, *SD* = 0.17; *M*_higher IAcc_ = 0.75, *SD* = 0.10), *t*(1189.28 = −52.70, *p* < 0.001, *r* = 0.14), but they did not differ with respect to age at both measurement time points [t1: Age_lower IAcc_: *M* = 8.39 years, *SD* = 0.94; Age_higher IAcc_: *M* = 8.35 years, *SD* = 0.96; *t*(1444) = 0.75, *p* = 0.45; t2: Age_lower IAcc_: *M* = 9.10 years, *SD* = 0.91, Age_higher IAcc_: *M =* 9.15 years, *SD* = 0.93; *t*(1450) = *−0.69,p* = 0.49].

**Table 1:** Intercorrelations of and descriptive statistics for the assessed variables at measurement points t_1_ and t_2_.

	1.	2.	3.	4.	5.	6.	7.	8.	9.
1. Interoceptive Accuracy t_1_									
2. IGT t_1_	−0.01								
3. DoG sweets t_1_	0.05	0.04							
4. DoG toys t_1_	0.04	0.01	0.39^**^						
5. Interoceptive accuracy t_2_	0.32^**^	0.04	0.01	−0.01					
6. IGT t_2_	0.00	0.27^**^	0.00	0.03	0.05				
7. DoG sweets t_2_	0.02	0.01	0.22^**^	0.17^**^	0.04	0.02			
8. DoG toys t_2_	0.03	0.03	0.24^**^	0.28^**^	0.01	0.06^**^	0.31^**^		
9. Age t_1_	−0.02	0.07^**^	0.09^**^	0.06^*^	0.05^*^	0.07^**^	0.03	0.00	
Mean	0.55	32.91	1.42	1.38	0.56	34.41	1.68	1.60	8.37
SD	0.26	6.22	0.71	0.76	0.24	6.92	0.57	0.64	0.94
Min-Max (sample)	0–0.98	13–58	0–2	0–2	0–1	12–60	0–2	0–2	6.23–11.33
Min-Max (theoretical)	0–1	0–60	0–2	0–2	0–1	0–60	0–2	0–2	

IGT, Child version of the Iowa Gambling Task; DoG, delay of gratification; period between t1 and t2 = approx. 1 year. *N* = 1,285–1,454.

* *p* ≤ 0.05;

** *p* ≤ 0.01.

### 3.1. Interoceptive accuracy and performance on the IGT

**Figure 1:**
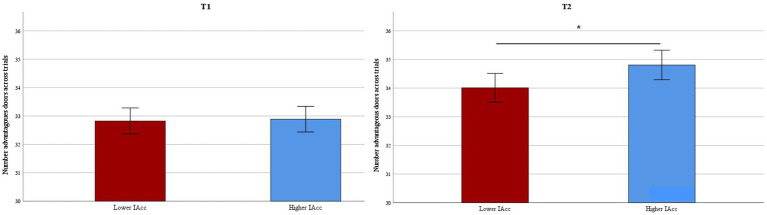
IGT performance: Mean number of advantageous choices (i.e., doors C and D) across trials in children with higher vs. lower interoceptive accuracy at t1 and t2 (error bars represent the 95% confidence interval). *p ≤ 0.05.

**Figure 2:**
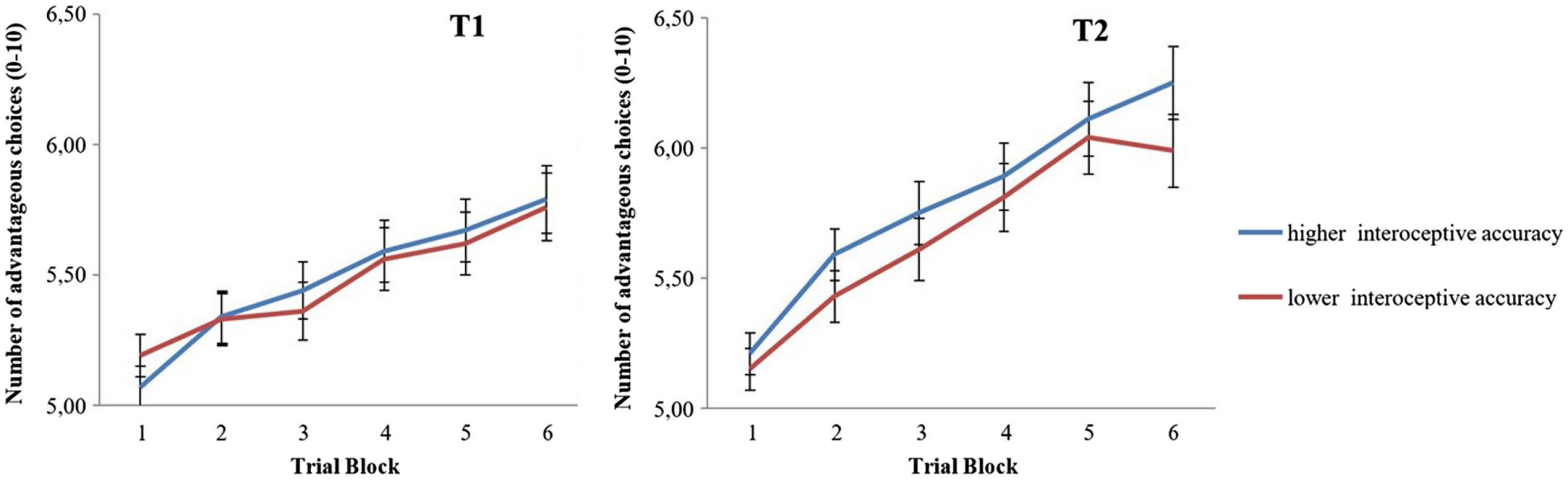
IGT performance: Number of advantageous choices (i.e., doors C and D), over 6 blocks of 10 trials each, in children with lower vs. higher interoceptive accuracy at t1 and t2 (error bars represent the 95% confidence interval).

The two ANOVAs for the IGT at t1 and t2, respectively, revealed that learning across trials was evident in both groups of Interoceptive Accuracy, indicated by a significant main effect of Trial Block at t1, *F*(4.84, 6924.98) = 53.23, *p* ≤ 0.01, η^2^ = 0.04, and at t2, *F*(4.67, 6645.87) = 115.76, *p* ≤ 0.01, η^2^ = 0.08. Moreover, at t2, children with higher interoceptive accuracy selected more advantageous doors than did children with lower interoceptive accuracy, indicated by a significant main effect of Interoceptive Accuracy, *F*(1, 1,422) = 4.73, *p* = 0.03, η^2^ < 0.01. This, however, was not yet the case at t1, *F*(1, 1,432) = 0.04, *p* = 0.84, η^2^ < 0.01 (see Figure 1). The interaction between Interoceptive Accuracy and Trial Block was not significant at t1, *F*(4.84, 6924.98) = 1.06, *p* = 0.38, η^2^ < 0.01, or at t2, *F*(4.67, 6645.87) = 1.32, *p* = 0.25, η^2^ < 0.01, indicating that higher vs. lower Interoceptive Accuracy did not significantly influence children’s progress of learning across IGT trials (see Figure 2). Subsequent analyses across distinct age groups (for descriptive statistics see Supplementary Table 1) indicated that all children, independent of age and interoceptive abilities, were able to learn to opt for more advantageous choices over the course of trials (see Supplementary material). Furthermore, in the middle age group (7.84–8.88 years) interoceptive accuracy was found to positively influence this learning process, but not the overall performance in the IGT (see Supplementary Figure 1); this effect was absent in the younger and older group.

### 3.2. Interoceptive accuracy and performance on the DoG task

**Figure 3:**
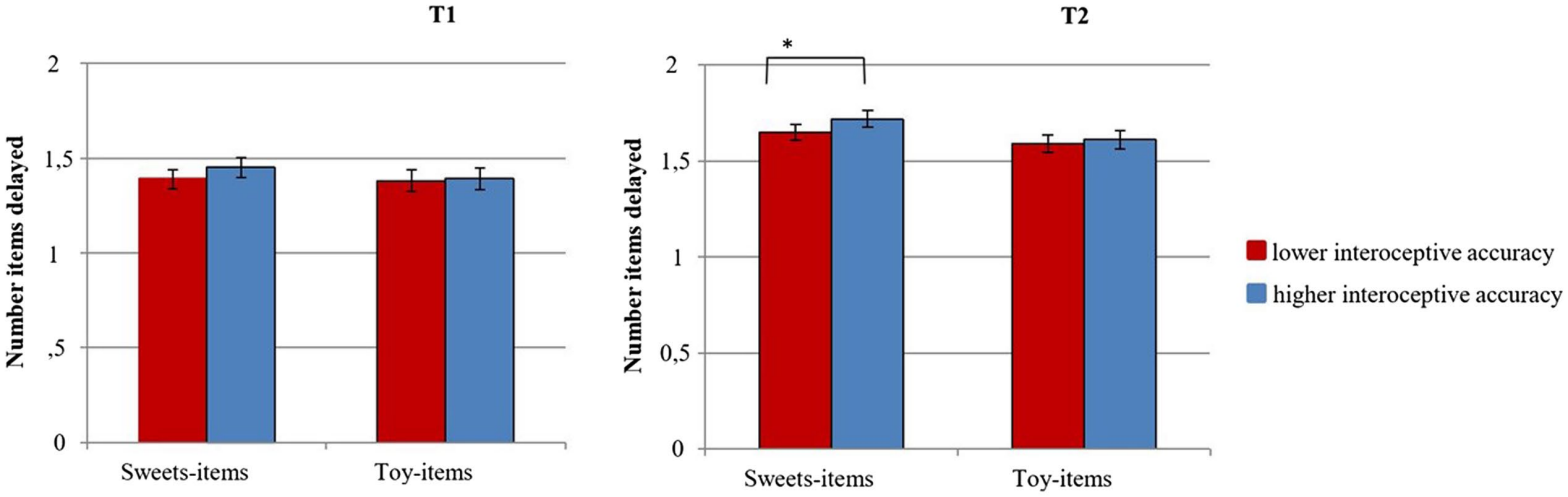
DoG performance: Number of sweets-items and toy-items delayed in children with lower vs. higher interoceptive accuracy at t1 and t2 (error bars represent the 95% confidence interval). *p ≤ 0.05.

The two MANOVAs at t1 and t2, respectively, for the DoG task revealed no significant main effect of Interoceptive Accuracy on the number of sweets-items or toy-items delayed at t1, *Pillai’s trace*, *V* = 0.002, *F*(2, 1,345) = 1.04, *p* = 0.35, η^2^ < 0.01 or at t2, *V* = 0.004, *F*(2, 1,379) = 2.57, *p* = 0.077, η^2^ < 0.01. Separate *post-hoc* univariate ANOVAs on the outcome variables revealed a significant main effect of Interoceptive Accuracy at t2, but only for sweets-items delayed, *F*(1, 1,380) = 5.15, *p* = 0.02, η^2^ < 0.01, and not for toy-items delayed, *F*(1, 1,380) = 0.52, *p* = 0.47, η^2^ < 0.01 (see Figure 3). Subsequent repetition of the analysis for distinct age groups at t_1_ revealed no significant effect of Interoceptive Accuracy on either of the age groups (see Supplementary material).

### 3.3. Longitudinal prediction of IGT and DoG performance by interoceptive accuracy

Regressing decision-making performance at t2 on Interoceptive Accuracy at t1 (lower versus higher), controlling for decision-making performance at t1 as well as for age, did not reveal a significant incremental effect of Interoceptive Accuracy on any of the three decision-making measures, IGT at t2 (number of advantageous doors selected): *B* = −0.25, *SE B* = 0.36, *p* = 0.49; DoG at t2 (sweets-items delayed): *B* = 0.01, *SE B* = 0.03, *p* = 0.79; DoG at t2 (toy-items delayed): *B* = 0.03, *SE B* = 0.03, *p* = 0.39 (see Table 2).

**Table 2:** Summary of the multiple-regression analyses for the decision-making measures.

*Variable*	*B*	*SE B*	*β*
**IGT at t2**			
Step 1			
Constant	23	1.82	
Age t1	0.30	0.19	00.04
IGT t1	0.27	0.03	00.24^**^
Step 2			
Constant	23.41	1.92	
Age t1	0.30	0.19	00.04
IGT t1	0.27	0.03	00.24^**^
Interoceptive accuracy t1	−0.25	0.36	−0.02
**DoG (sweets-items) at t2**			
Step 1^a^			
Constant	1.34	0.14	
Age t1	0.01	0.02	00.01
DoGSweets t1	0.18	0.02	00.22^**^
Step 2^a^			
Constant	1.33	0.15	
Age t1	0.01	0.02	00.01
DoGSweets t1	0.18	0.02	00.22^**^
Interoceptive accuracy t1	0.01	0.03	00.01
**DoG (toy-items) at t2**			
Step 1^b^			
Constant	1.32	0.15	
Age t1	−0.01	0.02	−0.01
DoGToys t1	0.24	0.02	00.28^**^
Step 2^b^			
Constant	1.27	0.16	
Age t_1_	−0.01	0.02	−0.01^**^
DoGToys t1	0.24	0.02	00.28^**^
Interoceptive accuracy t_1_	0.03	0.03	00.02^**^

IGT, Child version of the Iowa Gambling Task; DoG, Delay of gratification.

a R^2^ = 0.06 for Step 1, ΔR^2^ = 0.06 for Step 2.

b R^2^ = 0.08 for Step 1, ΔR^2^ = 0.08 for Step 2.

** *p* < 0.001.

## 4. Discussion

The aim of the present study was to investigate whether 6- to 11-year-old children with higher interoceptive accuracy (assessed by a Heartbeat-Perception Task; Schandry, 1981) show better decision-making performance (in terms of forgoing short-term benefit for long-term profit) than do children with lower interoceptive accuracy. Decision-making ability was assessed at two measurement time points (t1/t2, about 1 year apart) by two tasks that differed with respect to the time that children needed to wait for rewards as well as to the certainty with which rewards were obtained: a child-version of the IGT (i.e., Hungry-Donkey task; Crone and van der Molen, 2004) and a DoG task (with 2 sweets-items and 2 toy-items). Moreover, we studied whether children’s interoceptive accuracy would predict their later decision-making performance.

We found that first, across the 60 IGT trials, children in both interoceptive accuracy groups learned to select the advantageous doors more often. Additionally, children with higher (as compared to lower) interoceptive accuracy performed better in the IGT at t_2_, overall choosing for more advantageous and less risky options at this time point; this is at least partially consistent with our hypotheses. As the groups did not differ in terms of age, this difference cannot be attributed to a developmental advantage. However, neither at t_1_ nor at t_2_, we found evidence that the magnitude of increase of advantageous choices across trials was influenced by children’s interoceptive accuracy. Further subsequent comparisons of age groups at t_1_ revealed that all children, independent of age and interoceptive abilities were able to learn to opt for more advantageous choices in the IGT task. Additionally, this learning process was influenced by interoceptive accuracy, but only in the middle age group. Age group further influenced IGT performance, but not interoceptive accuracy.

In contrast to our hypothesis, correlations between the distinct variables were mostly low or non-significant. Furthermore, children with higher (as compared to lower) interoceptive accuracy did not delay more items in the DoG task, neither in general, nor across age groups. A post-hoc analysis revealed more delayed items at t_2_ in children with higher interoceptive accuracy, but only when items were food-specific. Unexpectedly, higher versus lower interoceptive accuracy did not predict 1-year-changes in rank percentage of decision-making performance.

The present IGT-results at t2 match the few existing studies showing that higher interoceptive accuracy is related to advanced IGT performance in healthy adults (Werner et al., 2009; Wölk et al., 2014). This is mainly in line with the SMH, which stresses the role of somatic-marker signals in guiding decision-making processes (Damasio, 1994, 1996). Thus, the present study is the first to indicate that already in middle childhood, better access to somatic feedback, in terms of enhanced interoceptive accuracy measured by cardiac-perception, seems to be associated with an enhanced ability to forgo short-term benefit for long-term profit. In line with findings of Werner et al. (2009) in adults, we did not find a significant interaction between interoceptive accuracy and IGT trial block, indicating that although children with higher interoceptive accuracy showed a better overall performance, both groups learned to choose more advantageously over the course of IGT trials. Furthermore, the relatively small effect sizes indicate that much of the variance in decision making is accounted for by variables other than interoceptive accuracy. Thus, although bodily feedback influences decision making, it may not be essential or their relation be mediated by factors not taken into account in the present study. Yip et al. (2020) proposed emotional intelligence as a moderator of the link between bodily responses and decision-making, such that adults with lower emotional intelligence tend toward a maladaptive association of bodily arousal and risky behavior. Accordingly, in children (aged 8–12) emotional intelligence is thought to contribute to IGT performance (Li et al., 2020). Consistently, there is evidence for a positive association between cardiac interoceptive accuracy and interpersonal emotional intelligence (Koch and Pollatos, 2014a) as well as emotion regulation (Opdensteinen et al., 2021). This is in line with the finding that interoceptive accuracy predicts aversion to loss in a gambling task in adults (Sokol-Hessner et al., 2015). Furthermore, it remains possible that in the IGT task, children and adults use forms of bodily feedback other than cardiac interoceptive accuracy. Decision making is probably supported by different systems being active to varying degrees, depending on individual and situational characteristics (Frijda, 2005; Knutson and Bossaerts, 2007; Laird, 2007; Dunn et al., 2010). Thus, future studies would benefit from adopting individual difference approaches to figure out for which individuals decision-making processes are positively influenced by bodily feedback in contrast to other information-processing mechanisms (Wölk et al., 2014; Moccia et al., 2021; Avcu Meriç and Sönmez, 2022). The role of individual differences in this process might be of even further relevance, as interoceptive processes are thought to have extensive bilateral implications for mental health and chronic pain already in children (Hechler, 2021).

To date, studies on the role of somatic markers in decision making have exclusively focused on variants of the IGT, measuring decision-making patterns given incomplete information or ambiguous consequences, respectively. To our knowledge, our study is the first to indicate that children’s interoceptive accuracy is also related to decision-making ability in situations that are less complex and ambiguous than the IGT, differing in the time that children need to wait for rewards and in the certainty with which rewards are obtained (i.e., in DoG tasks). However, we found children with higher interoceptive accuracy to show a stronger ability to delay gratification only for sweets-items, but not for toy-items. This finding is particularly interesting given the role of interoception in the regulation of eating and body weight (e.g., Pollatos et al., 2008; Ainley and Tsakiris, 2013; Herbert et al., 2013; Klabunde et al., 2013; Herbert and Pollatos, 2014; Young et al., 2017). In general, an accurate heartbeat perception is linked to a more finely-tuned behavioral self-regulation according to one’s bodily needs (Herbert et al., 2007). For example, eating-disordered or overweight individuals exhibit lower interoceptive accuracy than do normal-weight individuals (Herbert and Pollatos, 2014). This was also true for overweight children in the present sample, who exhibited a lower interoceptive accuracy than did normal-weight children at t2 (Koch and Pollatos, 2014b). Furthermore, overweight children tend to have difficulties in delaying gratification, especially when it is food-specific and palatable (Bonato and Boland, 1983). Seeyave et al. (2009) found that, among other factors, the ability to delay gratification in young children (age 4) is related to the likelihood of overweight in adolescence. Thus, the present results point to a possible link between lower interoceptive accuracy, difficulties in delaying food-specific gratification, and the development of overweight. However, these interesting initial findings need to be interpreted with caution because effect sizes were small and only post-hoc analyses turned out to be significant at an alpha-level of 0.05. Additionally, further analyses in the present study indicated that in contrast to our expectations, later decision-making abilities are *not* predicted by early interoceptive accuracy. Further studies are needed to verify those first explorative results examining the relations of these three variables during development in more detail.

In general, the associations between interoceptive accuracy and the two decision-making measures turned significant only at t2 when children were 7 to 11 years old, which might point to a developmental strengthening of the association over time. This was partially reflected by the analysis across age groups: in children of 7–8 years of age, interoceptive accuracy influenced the learning across IGT trials, but not the overall outcome. These results suggest a developmental change in the way children utilize bodily feedback in decision-making processes. However, no significant developmental change was found in the number of delayed items across age groups. During middle childhood, interoceptive accuracy as well as decision-making ability are not yet finally developed (Prencipe et al., 2011; Koch and Pollatos, 2014a), and there is evidence that bodily responses influence decision-making ability more strongly when interoceptive ability increases (Dunn et al., 2010). Thus, with progress in development of both abilities as well as with a consolidation of their linkage based on learning processes, the association between interoceptive accuracy and decision making probably gets increasingly established. However, the absence of similar findings in the youngest as well as the oldest age group indicates that this development is not linear and likely to be influenced by other factors in a complex system. In adults, improvement of interoceptive abilities after a training interval was further correlated with a shift toward rational decision making (Sugawara et al., 2020). Furthermore, the fact that significant associations only occurred at t2 could be taken to indicate that the age of 6 to 7 years might be crucial for consolidating the link between interoceptive accuracy and decision making. This is supported by the difference in interaction of interoception and decision making in the IGT between the youngest and middle-aged group. However, this speculative assumption needs to be further confirmed by future studies examining age effects in more detail. Further studies are required in order to resolve the developmental timetable of interoceptive and decision-making abilities and the role of further factors accompanying the development.

In contrast to our expectations, interoceptive accuracy did not influence subsequent 1-year-changes in rank percentage of decision-making performance, when controlling for age and decision-making performance at t1. This missing longitudinal relation could be explained first, by the influence of third variables (e.g., general cognitive ability or attention). Other studies in adults indicate that higher-order cognitive functions, such as working memory are required in order to mediate decision-making in response to somatic cues (Hinson et al., 2002). If so, higher interoceptive accuracy would simply occur as an epiphenomenon of enhanced decision-making performance, rather than being a preceding factor. Alternatively, interoceptive accuracy might start to influence the subsequent development of decision making only later in development, which would agree with the finding that cross-sectional associations did not occur until t2, when children were aged 7 to 11 years. Furthermore, interoceptive accuracy turned out to be only moderately stable between measurement time points. Thus, assuming that concurrent levels of interoceptive accuracy influence decision making, this finding would provide an additional explanation for the missing longitudinal relationship between both variables. Apparently, interoceptive accuracy cannot yet be seen as a stable trait during middle childhood; interestingly, neither can be decision-making (Smith et al., 2012; Li et al., 2020). It would be interesting for future studies to examine longitudinal relations throughout and later in development, with an assumingly increasing stability of interoceptive accuracy and decision making. Finally, within the 1-year interval of our study, decision making had a limited possibility to change. Thus, future studies would benefit from exploring longitudinal relations of variables over a longer time span.

### 4.1. Study limitations

The present study is the first to examine the relations between interoceptive accuracy and decision making in children at 6-11 years of age. Its strengths include the examined age group, the assessment of interoceptive accuracy in a large unselected sample of over 1.400 children, the use of a DoG task in addition to the IGT, and the longitudinal perspective across a 1-year period. However, the study has some limitations that need to be acknowledged and that provide directions for future research.

The Heartbeat-Perception Task applied in the present study is commonly used in the assessment of interoceptive accuracy (e.g., Ehlers et al., 2000; Eley et al., 2004; Pollatos et al., 2008; Dunn et al., 2010), and it has been successfully used with children (e.g., Koch and Pollatos, 2014a,b). However, by treating interoceptive accuracy as a dichotomous variable, we applied a rather coarse measure. Given the explorative nature of our research questions and the rather small expected effect sizes, we aimed to maximize the statistical power of our analyses by comparing two equally-sized groups of children that differed significantly with regard to their interoceptive accuracy. A further concern could be that there are a number of factors that might influence the results of Heartbeat-Perception Tasks, such as attentional processes or people’s beliefs and expectancies about their heart rates (Knapp et al., 1997; Wiens and Palmer, 2001; Knapp-Kline and Kline, 2005). Nevertheless, different Heartbeat-Perception Tasks lead to congruent results concerning effects of interoception on emotions (Katkin et al., 2001; Pollatos et al., 2005; Wiens, 2005; Pollatos et al., 2007), or concerning localization of relevant brain structures activated during heartbeat perception (Critchley et al., 2004; Pollatos et al., 2007), which supports the validity of such tasks in detecting processes involved in interoception. It might, however, be argued that the Heartbeat-Perception Task is not perfectly valid for children with limited ability to count precisely. This concern is further amplified as no time estimation task was performed as a control condition in order to avoid contamination by simply counting seconds (Desmedt et al., 2020). However, the estimation of heartbeats based on time interval estimation and respective calculations based on prior knowledge is more likely to be a problem in adult rather than in child populations. Furthermore, the Heartbeat-Perception Task was applied in children aged around 8–11 years before without additional control by a time estimation task (Eley et al., 2004; Georgiou et al., 2015). Nonetheless, alternative methods might assess interoceptive accuracy more reliably in children: the jumping jack paradigm is a tool that was designed to assess interoceptive accuracy in preschool-aged children to overcome such methodological limitations (Schaan et al., 2019; Opdensteinen et al., 2021); future research might benefit from sensibly choosing the task and control mechanisms for the assessment of interoceptive accuracy in order to provide even more reliable results. Additionally, future studies might investigate the contribution of cognitive factors such as working memory and their development in the Heartbeat-Perception Task in children in order to further validate the method and properly understand the relationship between the contributing factors.

It could also be criticized that, although both the IGT and the DoG task have been designed in order to mimic real-life decision making, it remains unclear whether results are transferable to a less artificial environment. Thus, future studies would benefit from examining interoceptive accuracy in relation to real-life decision making. Moreover, although we applied a longitudinal design, we only had two waves of data with a distance of a single year. Thus, we cannot completely rule out that task familiarity effects might have occurred. However, given the high complexity of the IGT, it seems very unlikely that children remembered the win/loss contingencies for the door choices, in particular because children were not informed about which doors were advantageous after the first test session.

Our findings indicated that the relation of interoceptive accuracy and decision making may be stronger at t2 (i.e., when children are 7 to 11 years old) than at t1 (i.e., about 1 year earlier). However, it would seem premature to make assumptions concerning a developmental framework on the basis of only two time points in children in this broad age range. In general, correlations between interoceptive accuracy, decision making, and age were low or non-existent, and extensively comparing single age groups was beyond the scope of the current paper.

Yet, while the examined age group can be considered as a strength of this study on the one hand, the age range (6 or 7–11, respectively) is rather broad in terms of development on the other hand, rendering the comparison of children at very different developmental stages rather delicate. While the question of the developmental trajectory of interoceptive abilities throughout childhood remains largely unresolved (Murphy et al., 2017), preschool children (aged 4–6) appear to become more sensitive or even oversensitive toward heart rate change with increasing age (Schaan et al., 2019). Additionally, decision-making abilities are thought to develop with age throughout childhood and adolescence (e.g., Crone and van der Molen, 2007), but not necessarily monotonously (Smith et al., 2012; Li et al., 2020). Thus, future studies might benefit from examining the developmental timetable of interoceptive and decision-making abilities in more detail by comparing single age groups and investigate the association over a longer time span as well as in older children, when both abilities have increasingly developed. Further analyses could also focus explicitly on the effect of age on the relationship between interoceptive accuracy and decision-making performance and the pivotal development at the age of 6–7 that was suggested in the present study.

Additionally, sex-specific differences were not taken into account in the present study, although gender might impact the state of development, particularly in young children. In a previous study with the same sample, Koch and Pollatos (2014a) revealed a male advantage on the Heartbeat-Perception Task. While Almy et al. (2018) did not find a significant effect of gender on IGT performance, other studies suggest that boys outperform girls on the IGT (Crone and van der Molen, 2007; Lensing and Elsner, 2018). Accounting for sex-specific differences exceeded the aims and scope of the present study; however, this might be an interesting subject to future research.

Furthermore, when evaluating the present findings, one should keep in mind that an alpha-level of 0.05 was applied for each hypothesis (for both tasks and measurement time points) and statistical power could be reduced in some cases due to violations of the prerequisites for the respective tests. Therefore, infering an overall relation between interoceptive accuracy and decision making during middle childhood can only be done with caution. Moreover, we cannot completely rule out that third variables, such as general cognitive ability, attention or emotional intelligence, might have affected the detected associations of interoceptive accuracy and decision making at t2. However, there neither is evidence that individuals with good cardiac perception exhibit superior cognitive performance, nor did the decision-making tasks show any associations to a fluid-intelligence measure in the present sample (Groppe and Elsner, 2014). Nevertheless, it would be interesting for future studies to examine the causality of effect in the association between cardiac perception and decision making, or the impact of potential moderators such as gender or cognitive factors.

## 5. Conclusion

Examining a large sample of children, our results for the first time indicate that individual differences in interoceptive accuracy relate to decision-making abilities in situations of varying complexity as early as in middle childhood, showing that already at the age of 7–11 years, the perception of and sensitivity to somatic feedback in form of cardiac cues may help in forgoing short-term reward for long-term profit, providing valuable contribution to the evidence on the SMH. Furthermore, the association of interoceptive accuracy and the delay of sweets-items might have implications for the regulation of body weight at a later age. These associations did not occur until the second measurement time point, which indicates a probable consolidation of the link between interoceptive accuracy and decision making over the course of development. However, effect sizes were rather small and results did not confirm that interoceptive accuracy could predict changes in rank percentage of decision making across a 1-year-period. Furthermore, the study design does not allow for causal inferences concerning relationships between variables. Thus, future studies are needed to validate these first findings and to investigate the exact role of somatic-marker information for decision making over the course of development in more detail. Such studies should examine the reported associations over a longer time-span with tests specifically adapted for children and should consider other potential intervening variables.

## Data availability statement

The raw data supporting the conclusions of this article will be made available by the authors on request, without undue reservation.

## Ethics statement

The studies involving human participants were reviewed and approved by Research Ethics Board at the University of Potsdam; Ministry of Education, Youth and Sports of the Federal State of Brandenburg. Written informed consent to participate in this study was provided by the participants’ legal guardian/next of kin.

## Author contributions

The original paper was written by OP, BE, and KG and updated and revised by KM. All authors contributed to the article and approved the submitted version.

## Funding

This research was supported by a grant from the Deutsche Forschungsgemeinschaft (DFG; GRK 1668/1).

## Conflict of interest

The authors declare that the research was conducted in the absence of any commercial or financial relationships that could be construed as a potential conflict of interest.

## Publisher’s note

All claims expressed in this article are solely those of the authors and do not necessarily represent those of their affiliated organizations, or those of the publisher, the editors and the reviewers. Any product that may be evaluated in this article, or claim that may be made by its manufacturer, is not guaranteed or endorsed by the publisher.

## Abbreviations

DoG: Delay of gratification
IAcc: Interoceptive accuracy
IGT: Iowa gambling task
PFC: Prefrontal cortex
SMH: Somatic marker hypothesis
VM-PFC: Ventro-medial prefrontal cortex
