# Supplementary material for: Interoceptive accuracy is associated with benefits in decision making in children
Source: Front Psychol. 2023 Jan 19;13:1070037. doi: 10.3389/fpsyg.2022.1070037 (PMC9893641; doi:10.3389/fpsyg.2022.1070037)
Supplement: Supplementary file 1 [file Table_1.DOCX]

*Supplementary Material*

**Interoceptive accuracy is associated to benefits in decision making in children**

Olga Pollatos, Karla Mönkemöller*, Karoline Groppe, Birgit Elsner

**Correspondence:** Karla Mönkemöller, karla.moenkemoeller@uni-ulm.de

*Supplementary Table 1:* Descriptive statistics for the assessed variables across age groups

|  | Age group | | |
| --- | --- | --- | --- |
|  | 1 | 2 | 3 |
| N (% male) | 468 (47.65%) | 478 (46.44%) | 488 (50.00%) |
| Age Mean (SD) | 7.27 (0.36) | 8.37 (0.30) | 9.43 (0.42) |
| Age Min-Max | 6.37-7.83 | 7.84-8.88 | 8.88-11.33 |
| Interoceptive Accuracy Mean (SD) | 0.55 (0.27) | 0.54 (0.25) | 0.56 (0.25) |
| High interoceptive accuracy (%) | 52.14% | 46.03% | 51.23% |
| IGT Mean (SD) | 32.10 (5.85) | 33.15 (6.58) | 33.30 (6.12) |
| Sweet items delayed | 1.30 (0.76) | 1.42 (0.69) | 1.53 (0.64) |
| Toy items delayed | 1.31 (0.80) | 1.42 (0.75) | 1.41 (0.72) |

*Note:* IGT = Child version of the Iowa Gambling Task; age in years

*Summary of ANOVAs across age groups*

When repeating the ANOVAs on IGT performance for distinct age groups, significant main effects of Trial Block for all age groups (age group 1: F(5, 2339) = 13.69; p < .001, η² = .03); age group 2: F(5, 2380) = 20.69, p < .001, η² = .40; age group 3: F(5, 2430) = 20.61, p < .001 η² = .04) were found, suggesting that children of all ages are, in general, able to learn to opt for more advantageous doors over the course of trials. Additionally, in the middle age group, interoceptive abilities influenced this learning process over the course of trials, indicated by a significant interaction effect between Interoceptive Accuracy and Trial block (F(5, 2380) = 2.49, p = .029, η² = .005), but not the overall outcome of IGT performance (F(1, 476) = 0.86, p = .36). Neither Interoceptive Accuracy (age group 1: F(1, 466) = 1.65, p = 0.20; age group 3: F(1, 486) = 2.85, p = .092) nor the interaction with Trial Block (age group 1: F(5, 2330 = 0.34, p = 0.89; age group 3: F(5, 2430) = 1.71, p = .129) reached significance in younger or older children. Further post hoc analyses revealed that age group had a significant effect on IGT performance (i.e. number of advantageous doors; F(2, 1633), p = .015, η² = .005) but not on Interoceptive Accuracy (F(2, 1443) = 0.77, p = .46).


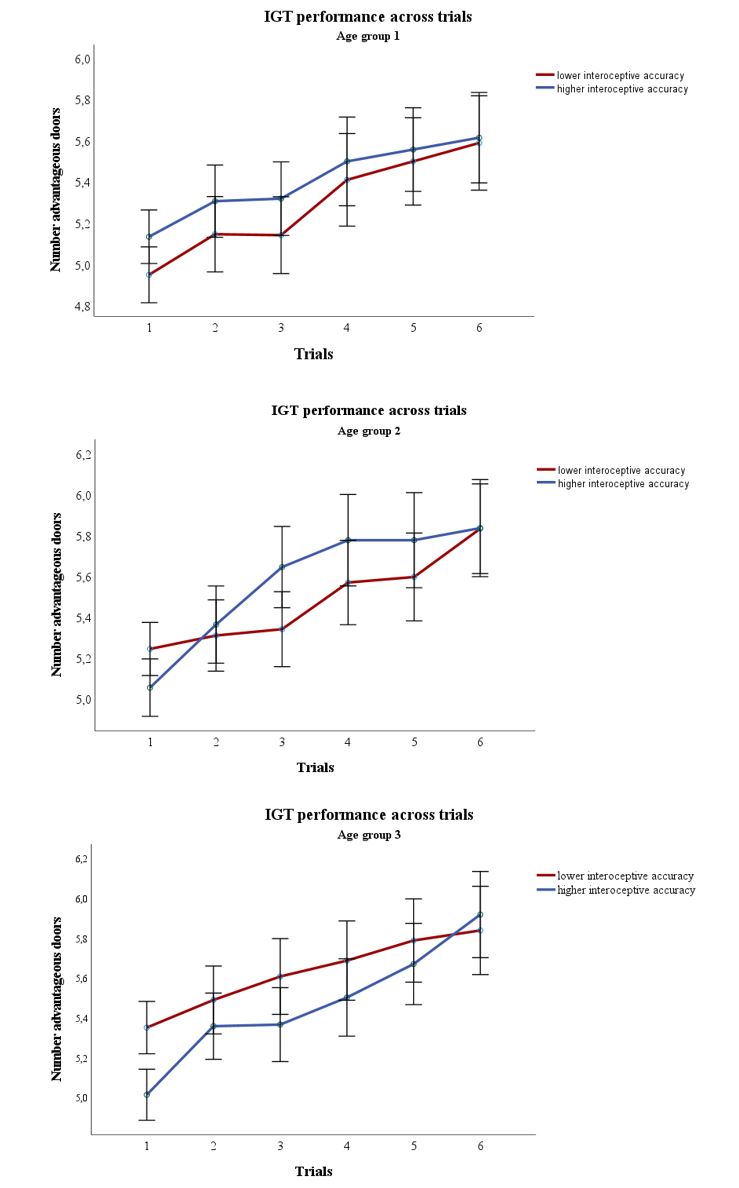


*Supplementary Figure 1*: IGT performance: Number of advantageous choices (i.e., doors C and D), over 6 blocks of 10 trials each, in children with lower versus higher interoceptive accuracy across age groups (error bars represent the 95% confidence interval

*Summary of MANOVAs across age groups*

For the youngest group, there was no significant difference in terms of delayed items between children with higher and children with lower interoceptive accuracy (*Pillai's trace*, V = 0.004, F(2, 438) = 0.86; p = .425, η² < .01). Post hoc univariate ANOVAs revealed no significant differences for sweet (F(1, 439) = 1.67; p=.197, η² <0 .01) or toy items (F(1, 439) = 0.07, p = .07, η² < .001) between the groups, respectively. For the middle group, differences did not reach significance either (*Pillai's trace*, V = 0.002, F(2, 450) = 0.38, p = .69,η² < .01). Neither did post hoc comparisons for sweet (F(1, 451) = 0.33, p = .57, η² < .01) or toy (F(1, 451) = 0.17, p = .69, η² < .001) items. Similar results were found in the oldest group: the analysis revealed no significant effect of Interoceptive Accuracy on the number of items delayed (*Pillai's trace*, V = 0.009, F(2, 451) = 1.94, p = .145, η² < .01). Post hoc univariate ANOVAs on sweet (F(1, 452) = 3.25, p = .07, η² < .01) and toy items (F(1, 451) = 0.00, p = .997, η² < .001) did not reach significance either.
